# Correction: Protection against *N. gonorrhoeae* induced by OMV-based meningococcal vaccines are associated with cross-species directed humoral and cellular immune responses

**DOI:** 10.3389/fimmu.2025.1697855

**Published:** 2025-09-12

**Authors:** Weiyan Zhu, Andreea Waltmann, Marguerite B. Little, Kristie L. Connolly, Kathryn A. Matthias, Keena S. Thomas, Mary C. Gray, Aleksandra E. Sikora, Alison K. Criss, Margaret C. Bash, Andrew N. Macintyre, Ann E. Jerse, Joseph A. Duncan

**Affiliations:** ^1^ Division of Infectious Diseases, University of North Carolina School of Medicine, Chapel Hill, NC, United States; ^2^ Department of Pharmacology, University of North Carolina School of Medicine, Chapel Hill, NC, United States; ^3^ Department of Microbiology and Immunology, Uniformed Services University, Bethesda, MD, United States; ^4^ Laboratory of Bacterial Polysaccharides, Division of Bacterial, Parasitic, and Allergenic Products, Center for Biologics Evaluation and Research, US Food and Drug Administration, Silver Spring, MD, United States; ^5^ Department of Microbiology, Immunology, and Cancer Biology, University of Virginia, Charlottesville, VA, United States; ^6^ Department of Pharmaceutical Sciences, College of Pharmacy, Oregon State University, Corvallis, OR, United States; ^7^ Vaccine and Gene Therapy Institute, Oregon Health & Science University, Beaverton, OR, United States; ^8^ Department of Medicine, Duke Human Vaccine Institute, Duke University School of Medicine, Durham, NC, United States

**Keywords:** *Neisseria gonorrhoeae*, vaccine, *Neisseria meningitidis*, outer membrane vesicle (OMV), correlates of protection

In the original article, an incorrect number was provided for National Institutes of Health grant number “U19-AI113170”. The correct number is “U19-AI144180”.

The original version of this article has been updated.

