# Supplementary material for: Protection against N. gonorrhoeae induced by OMV-based meningococcal vaccines are associated with cross-species directed humoral and cellular immune responses
Source: Front Immunol. 2025 Apr 11;16:1539795. doi: 10.3389/fimmu.2025.1539795 (PMC12021806; doi:10.3389/fimmu.2025.1539795)
Supplement: Supplementary file 1 [file DataSheet1.docx]

**Supplemental Figures and Figure Legends**

**Supplemental Figure 1**

**
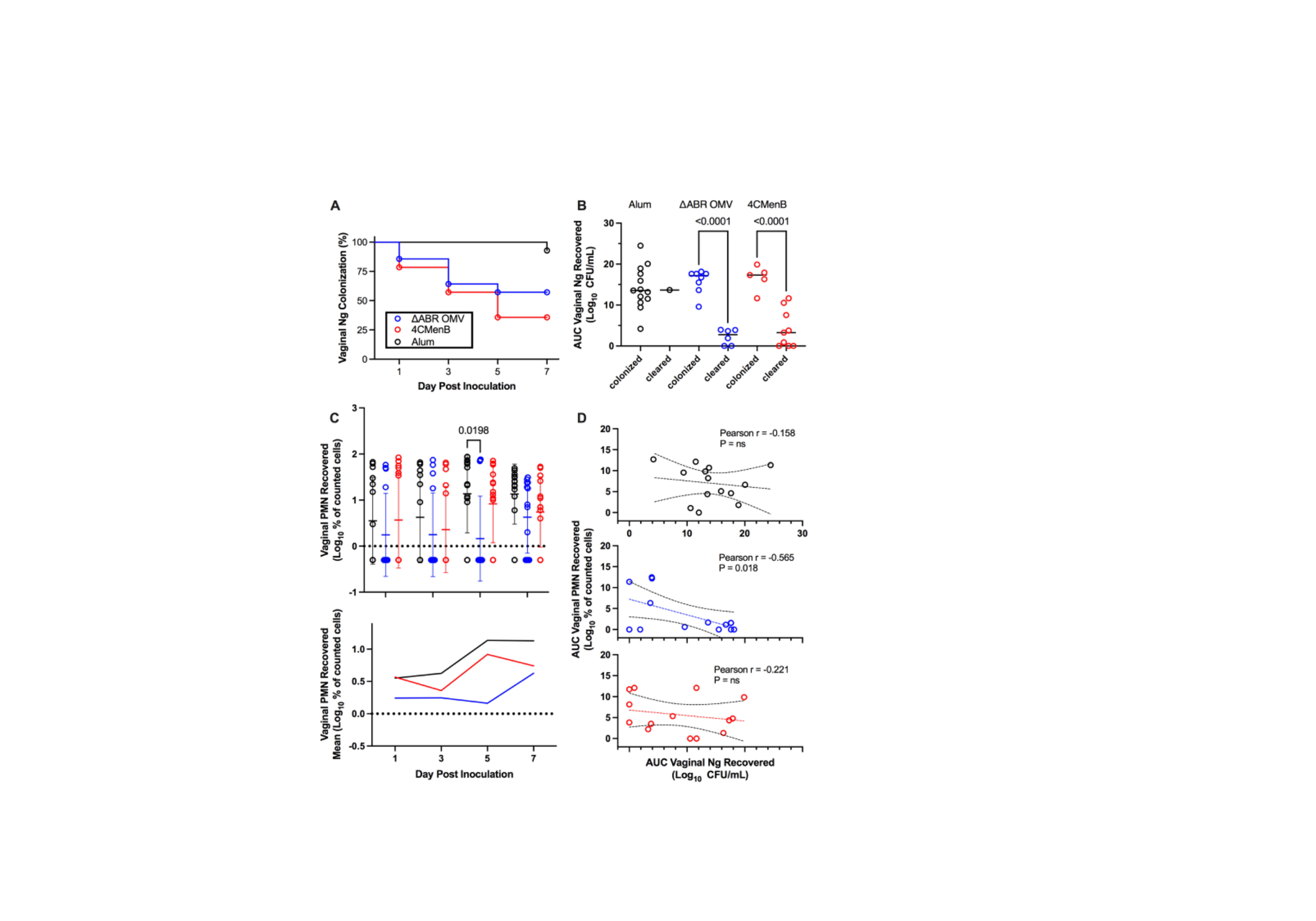
**

**Supplemental Figure 1: Experiment 1: Vaccination with MC58 ΔABR and 4CMenB enhance *N. gonorrhoeae* clearance from lower genital tract in mice.** The data from the first immunization/challenge experiment performed is presented in this figure. (A) Fraction of mice remaining infected over time with *N. gonorrhoeae* strain F62 after intravaginal inoculation are shown for mice vaccinated with Alum (adjuvant alone control), MC58 ΔABR OMV, and 4CMenB as indicated. Differences between infection persistence were compared using a log-rank (Mentel-Cox) test; *P* were 0.0254 or 0.0011 when MC58 ΔABR OMV and 4CMenB were compared to the Alum control. (B) Vaginal swab specimens were quantitatively cultured to determine the bacterial burden (number of CFU per milliliter) on days 1,3,5, and 7, the total *N. gonorrhoeae* bacterial burden (AUC vaginal *N. gonorrhoeae* recovered) for the infection course was determined for each individual mouse by taking the area under the curve of the log of the recovered CFU plotted against day post infection. The AUC are plotted for mice in each immunization group and groups are split into those mice that cleared infection by day 7 and those that had persistent infection at day 7. Statistical significance was performed using 2-way ANOVA followed by Bonferroni Post-hoc multiple comparisons to compare immunization groups. (C) Neutrophils (PMN) recovered on vaginal swabs collected on indicated days were quantified and plotted for individual mice in each vaccination group on each day (top panel) and as the mean for the group (bottom panel). (D) The total neutrophil recovery for the course of the infection was determined for each individual mouse by determining the area under the curve of the plotted recovered neutrophils over time and the AUC of Vaginal PMN recovery was plotted against the *N. gonorrhoeae* burden (AUC of Vaginal *N. gonorrhoeae* recovery) for each individual mouse, the Pearson Correlation Coefficient was determined for each immunized group of mice to assess for significant correlations between vaginal PMN and recovered bacteria.

Created in BioRender. Duncan, J. (2025) <https://BioRender.com/e26q650>

**Supplemental Figure 2**

**
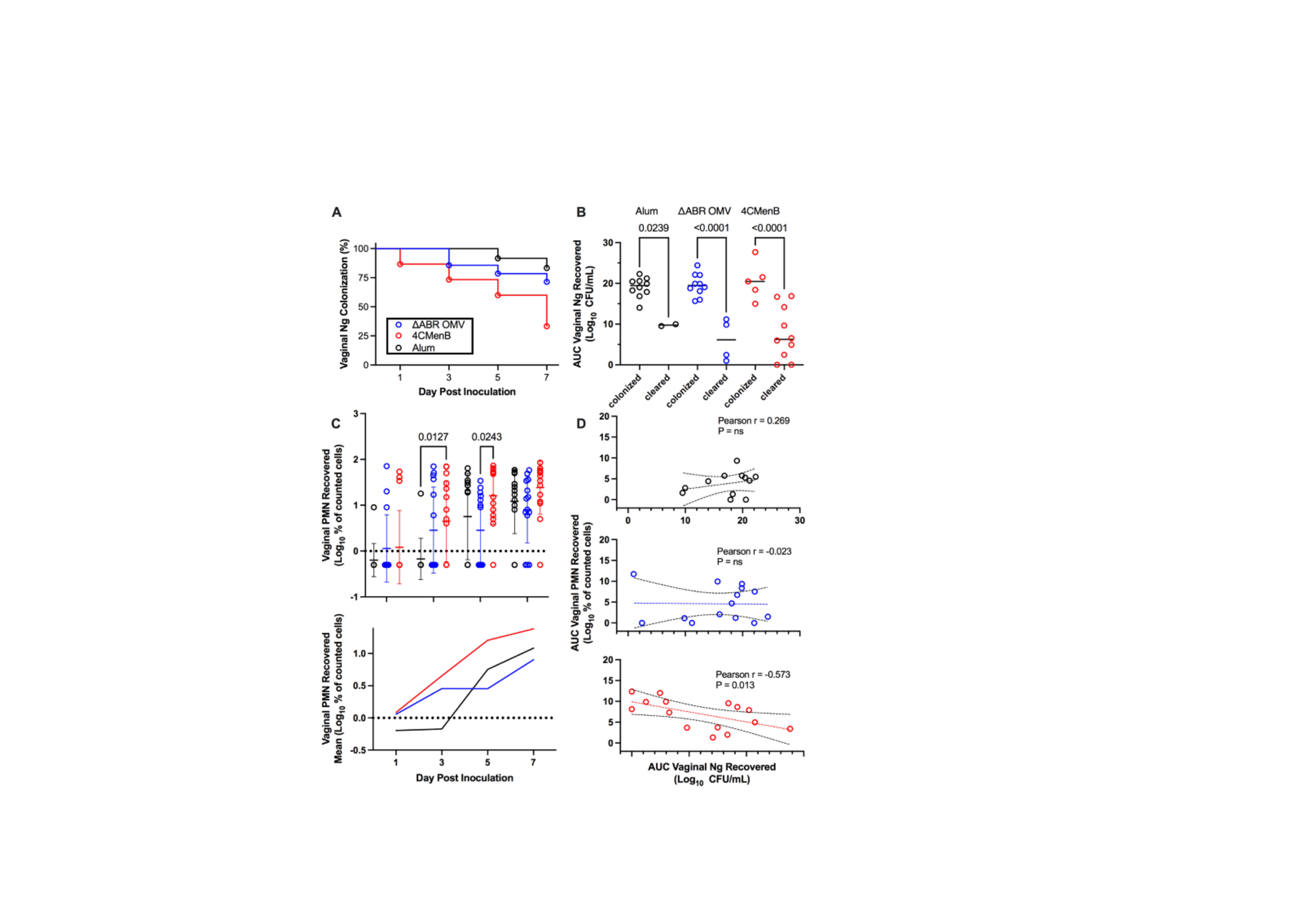
**

**Supplemental Figure 2: Experiment 2: Vaccination with MC58 ΔABR and 4CMenB enhance *N. gonorrhoeae* clearance from lower genital tract in mice.** The data from the second immunization/challenge experiment performed is presented in this figure. (A) Fraction of mice remaining infected over time with *N. gonorrhoeae* strain F62 after intravaginal inoculation are shown for mice vaccinated with Alum (adjuvant alone control), MC58 ΔABR OMV, and 4CMenB as indicated. Differences between infection persistence were compared using a log-rank (Mentel-Cox) test; *P* were 0.44 or 0.01 when MC58 ΔABR OMV and 4CMenB were compared to the Alum control. (B) Vaginal swab specimens were quantitatively cultured to determine the bacterial burden (number of CFU per milliliter) on days 1,3,5, and 7, the total *N. gonorrhoeae* bacterial burden (AUC vaginal *N. gonorrhoeae* recovered) for the infection course was determined for each individual mouse by taking the area under the curve of the log of the recovered CFU plotted against day post infection. The AUC are plotted for mice in each immunization group and groups are split into those mice that cleared infection by day 7 and those that had persistent infection at day 7. Statistical significance was performed using 2-way ANOVA followed by Bonferroni Post-hoc multiple comparisons to compare immunization groups. (C) Neutrophils (PMN) recovered on vaginal swabs collected on indicated days were quantified and plotted for individual mice in each vaccination group on each day (top panel) and as the mean for the group (bottom panel). (D) The total neutrophil recovery for the course of the infection was determined for each individual mouse by determining the area under the curve of the plotted recovered neutrophils over time and the AUC of Vaginal PMN recovery was plotted against the *N. gonorrhoeae* burden (AUC of Vaginal *N. gonorrhoeae* recovery) for each individual mouse, the Pearson Correlation Coefficient was determined for each immunized group of mice to assess for significant correlations between vaginal PMN and recovered bacteria.

Created in BioRender. Duncan, J. (2025) <https://BioRender.com/o33n522>

**Supplemental Figure 3**

**
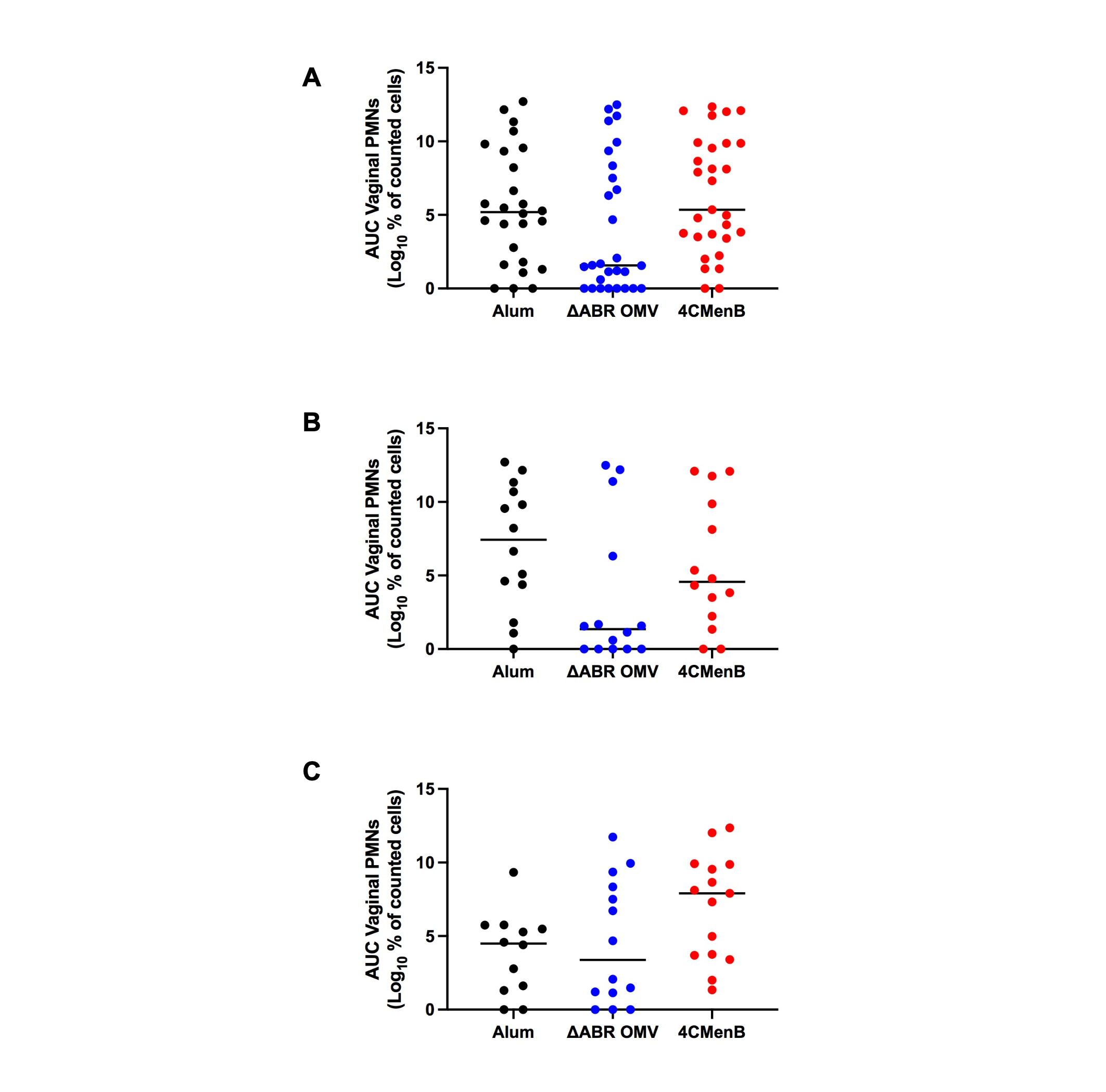
**

**Supplemental Figure 3: Total vaginal neutrophil influx during *N. gonorrhoeae* challenge in mice was not affected by the vaccination group.** Neutrophils (PMNs) collected from vaginal swabs on days 1, 3, 5, and 7 were quantified and plotted for individual mice in each group. The overall neutrophil influx throughout the infection was calculated for each mouse by determining the area under the curve (AUC) of the neutrophil counts over time, and the results were plotted by vaccination group (Alum: black, MC58 ΔABR: blue, and 4CmenB: red). Mean neutrophil influx between groups was compared using a one-way ANOVA followed by Tukey's multiple comparisons test, and no significant differences were found. Data are shown for combined experiments 1 and 2 (A), experiment 1 (B), and experiment 2 (C).

Created in BioRender. Duncan, J. (2025) <https://BioRender.com/z45s825>

**Supplemental Figure 4**

**
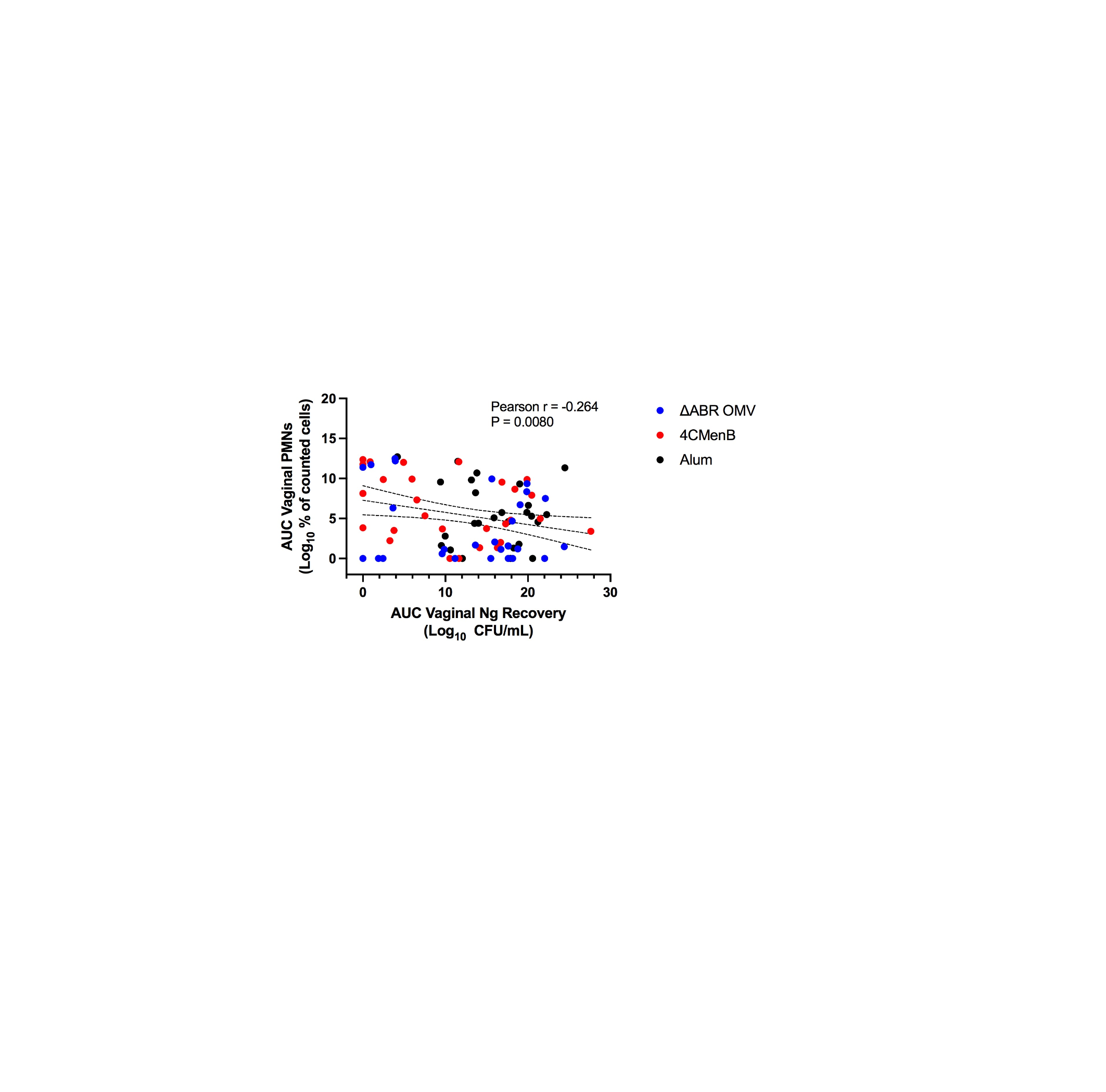
**

**Supplemental Figure 4:** T**he total neutrophil influx was inversely correlated to the total recovered *N. gonorrhoeae* CFU at the individual mouse level.** The total neutrophil recovery for the course of the infection, expressed as the area under the curve AUC of log_10_ (% of counted cells) recovered vaginal PMN over time, was plotted against the *N. gonorrhoeae* burden (AUC of log_10_ (CFU)) for each individual mouse. The Pearson Correlation Coefficient was determined for all mice to assess for significant correlations between vaginal PMN and recovered bacteria.

Created in BioRender. Duncan, J. (2025) <https://BioRender.com/x40d871>

**Supplemental Figure 5**

**
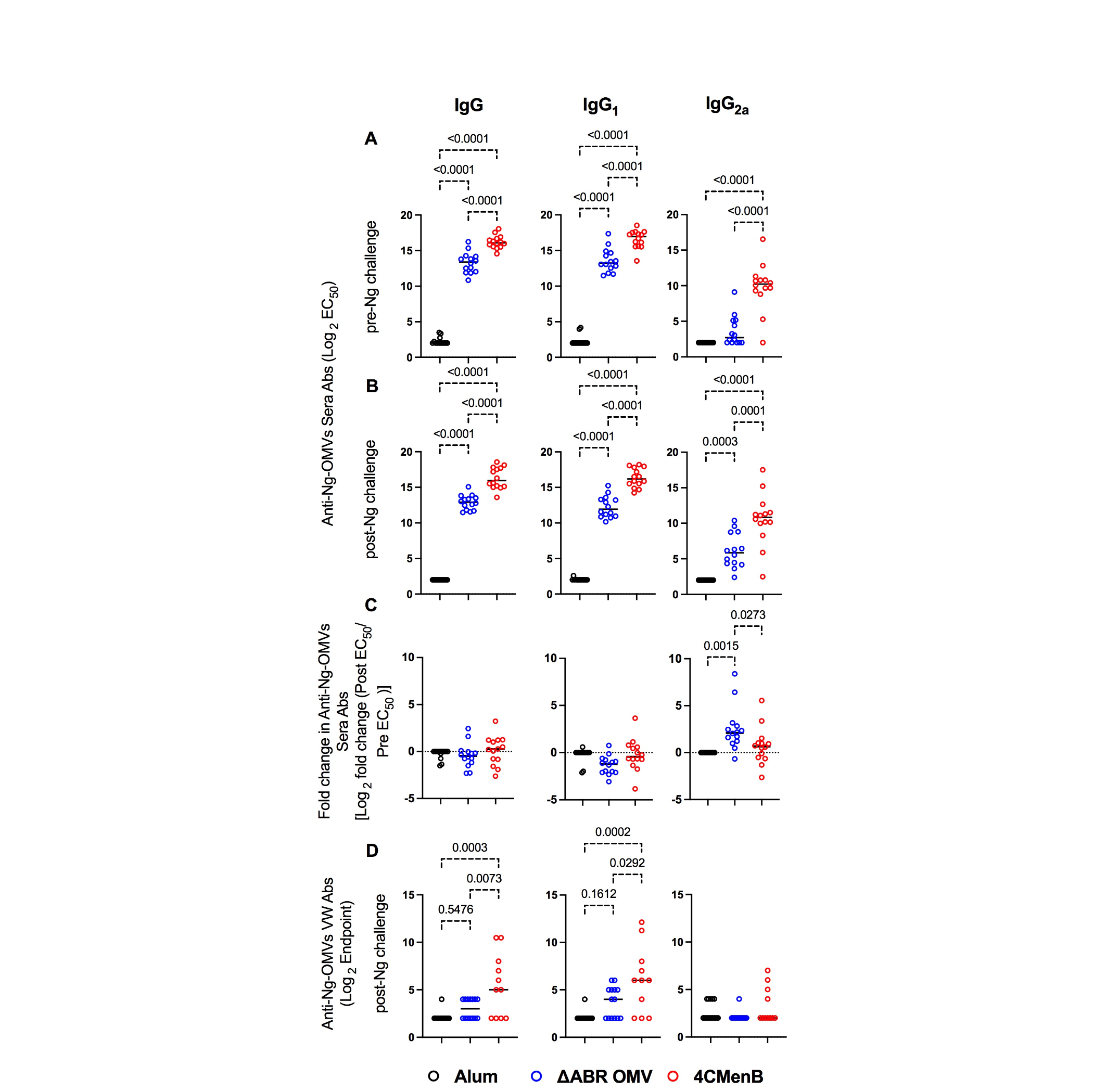
**

**Supplemental Figure 5: Experiment 1: Vaccination with MC58 ΔABR and 4CMenB elicited cross-reactive anti-Ng-OMV antibodies in serum and vaginal fluid.**  The data from the first immunization/challenge experiment performed is presented in this figure. *N. gonorrhoeae* OMV reactive antibody levels were measured by ELISA as described in Materials and Methods in sera collected at day 52, two weeks after final vaccination (A), and at terminal sera collection after *N. gonorrhoeae* challenge (B), and in terminal vaginal wash after *N. gonorrhoeae* challenge (D). The dilution of sera resulting in 50% maximal signal (EC50) and the greatest dilution of vaginal wash fluid resulting in signal above the baseline (Endpoint titer) were determined for total IgG, IgG1 or IgG2a isotype using immunoglobulin subclass specific secondary antibodies. (C) the fold change in each indicated *N. gonorrhoeae* OMV-directed immunoglobulin level between serum collected before challenge and after *N. gonorrhoeae* challenge was also determined. Levels from Alum (black), MC58 ΔABR (blue) and 4CMenB (Red) immunized mice are reported. Data were represented at log scale. Statistical significance was performed using one-way ANOVA followed by no paring Šidák’s multiple comparison test, with a single polled variance.

Created in BioRender. Duncan, J. (2025) <https://BioRender.com/b99z710>

**Supplemental Figure 6**

**
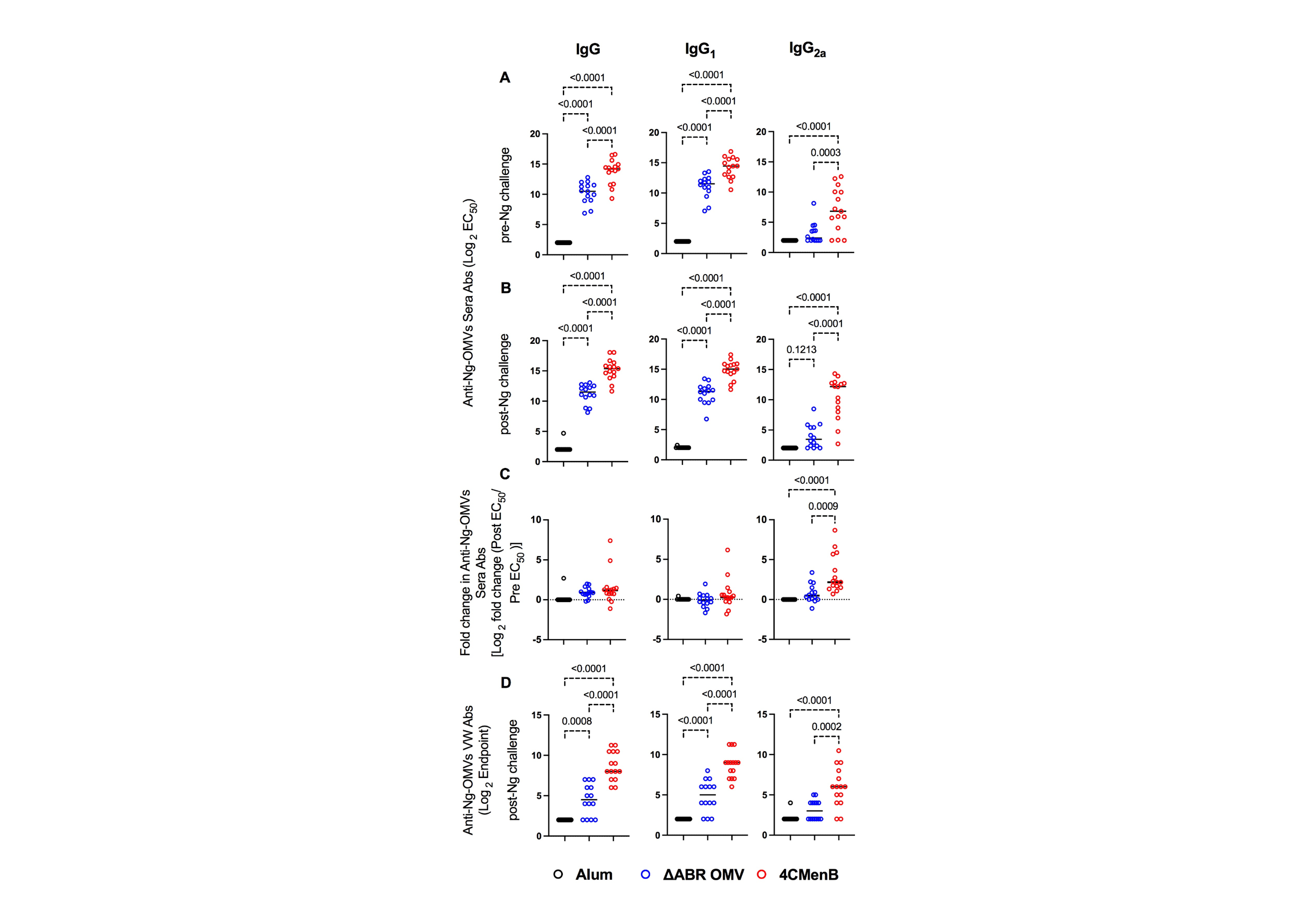
**

**Supplemental Figure 6: Experiment 2: Vaccination with MC58 ΔABR and 4CMenB elicited cross-reactive anti-Ng-OMV antibodies in serum and vaginal fluid.** The data from the second immunization/challenge experiment performed is presented in this figure. *N. gonorrhoeae* OMV reactive antibody levels were measured by ELISA as described in Materials and Methods in sera collected at day 52, two weeks after final vaccination (A), and at terminal sera collection after *N. gonorrhoeae* challenge (B), and in terminal vaginal wash after *N. gonorrhoeae* challenge (D). The dilution of sera resulting in 50% maximal signal (EC50) and the greatest dilution of vaginal wash fluid resulting in signal above the baseline (Endpoint titer) were determined for total IgG, IgG1 or IgG2a isotype using immunoglobulin subclass specific secondary antibodies. (C) the fold change in each indicated *N. gonorrhoeae* OMV-directed immunoglobulin level between serum collected before challenge and after *N. gonorrhoeae* challenge was also determined. Levels from Alum (black), MC58 ΔABR (blue) and 4CMenB (Red) immunized mice are reported. Data were represented at log scale. Statistical significance was performed using one-way ANOVA followed by no paring Šidák’s multiple comparison test, with a single polled variance.

Created in BioRender. Duncan, J. (2025) <https://BioRender.com/q82a988>

**Supplemental Figure 7**

**
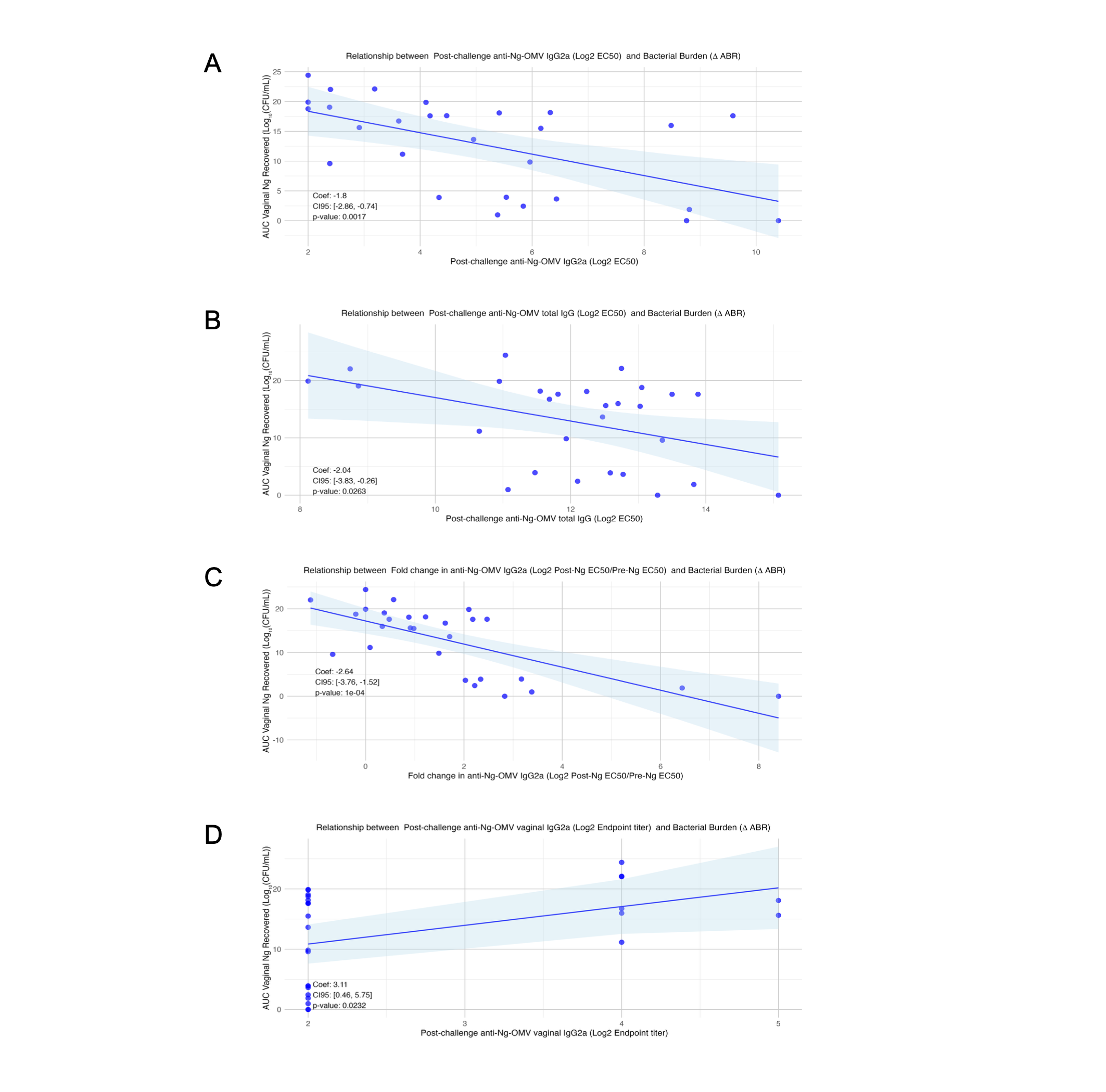
**

**Supplemental Figure 7: Linear regression analysis of vaginal *N. gonorrhoeae* burden and anti-*N. gonorrhoeae* immunoglobulins measured by ELISA.** The relationship between bacterial burden and antibody levels was assessed in the ΔABR group (blue) using linear regression in R. Regression coefficients (Coef), 95% confidence intervals (CI95, shown as shaded regions), and p-values are displayed for each panel. Each point represents data from an individual mouse. (A) Bacterial burden (*N. gonorrhoeae* recovered from vaginal swabs, AUC Log10 CFU/mL) was plotted against post-challenge anti-Ng-OMV IgG2a (Log2 EC50). (B) Bacterial burden (*N. gonorrhoeae* recovered from vaginal swabs, AUC Log10 CFU/mL) was plotted against post-challenge total IgG (Log2 EC50). (C) Bacterial burden (*N. gonorrhoeae* recovered from vaginal swabs, AUC Log10 CFU/mL) was plotted against the fold change in anti-Ng-OMV IgG2a (Log2 (Post-Ng EC50/Pre-Ng EC50)) (D) Bacterial burden (*N. gonorrhoeae* recovered from vaginal swabs, AUC Log10 CFU/mL) was plotted against post-challenge vaginal IgG2a endpoint titers (Log2). Created in BioRender. Duncan, J. (2025) https://BioRender.com/x12x064

**Supplemental Figure 8**

**
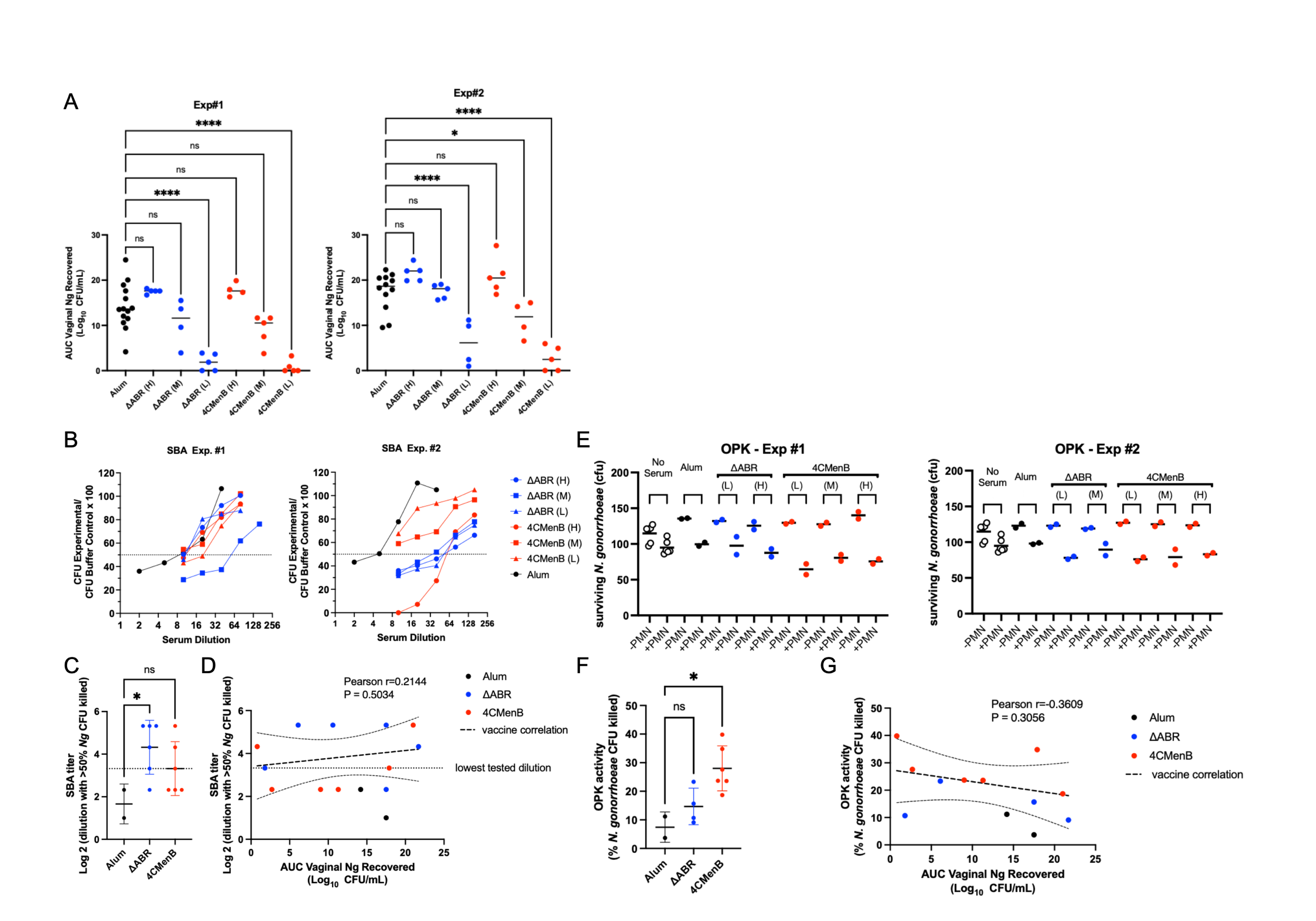
**

**Supplemental Figure 8: Serum bactericidal and opsonophagocytic activities against *N. gonorrhoeae* of pooled sera from MC58 ΔABR (blue) and 4CMenB (red) immunized mice are not correlated with clearance of *N. gonorrhoeae*.** Sera from immunized and N. gonorrhoeae challenged mice were pooled in groups based on the level of N. gonorrhoeae recovered during challenge, the AUC of recovered *N. gonorrhoeae* (Log10 CFU/mL) for each mouse in the pool is plotted **(A).** The bactericidal activity against *N. gonorrhoeae* strain F62 Serial dilutions of each pool was tested and the fraction of bacteria alive after treatment with serum and complement as described in materials and methods is plotted **(B)** The SBA titer of each pool (defined as the most diluted serum concentration to achieve >50% bacterial killing) is plotted by vaccine group **(C)** and against the mean bacterial burden during *N. gonorrhoeae* challenge for the pool of mice **(D)**  Opsonophagocytic killing assays against *N. gonorrhoeae* were performed as described in the materials and methods and surviving bacteria in each reaction with and without addition of PMN cells are plotted for no serum (control) and each serum pool **(E)**. The additional percent of bacteria killed for serum treated specimens compared to control is plotted for each serum pool according to the vaccine the mice received **(F)** and against the mean bacterial burden during *N. gonorrhoeae* challenge for the pool of mice. Linear regression analysis was performed in **(D)** and **(G)** to assess whether an association between activity and bacterial burden during infection was observed. Created in BioRender. Duncan, J. (2025) https://BioRender.com/r01j815

**Supplemental Figure 9**

**
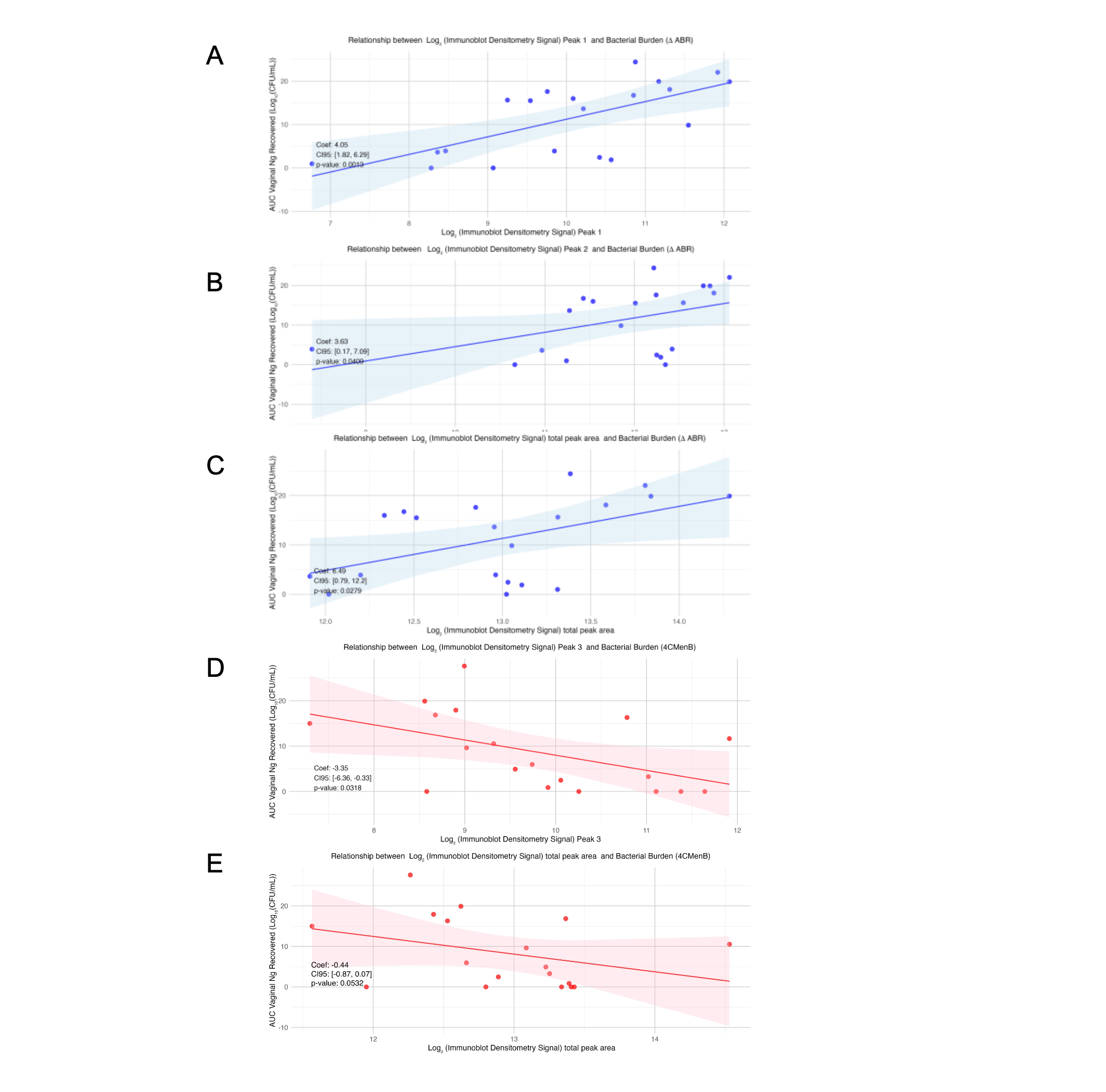
**

**Supplemental Figure 9: Linear regression analysis of vaginal *N. gonorrhoeae* burden and anti-*N. gonorrhoeae* immunoglobulins measured by immunoblot.** The relationship between bacterial burden and immunoblot densitometry signals measured in Fig 5A was assessed across two vaccine groups (ΔABR in blue and 4CMenB in red) using linear regression in R. Regression coefficients (Coef), 95% confidence intervals (CI95, shown as shaded regions), and p-values are displayed for each panel. Each point represents data from an individual mouse. For the ΔABR group (blue), bacterial burden (*N. gonorrhoeae* recovered from vaginal swabs, AUC Log10 CFU/mL) was plotted against the densitometry signals for Peak 1- 85kDa band in Fig 5A (A), Peak 2- 66 kDa band in Fig 5A (B), and the total peak area (C). In the 4CMenB group (red), bacterial burden was plotted against with densitometry signals for Peak 3- 37 kDa band in Fig 5A (D) and showed a trend toward a negative correlation with the total peak area (E). Created in BioRender. Duncan, J. (2025) https://BioRender.com/u29l787

**Supplemental Figure 10**


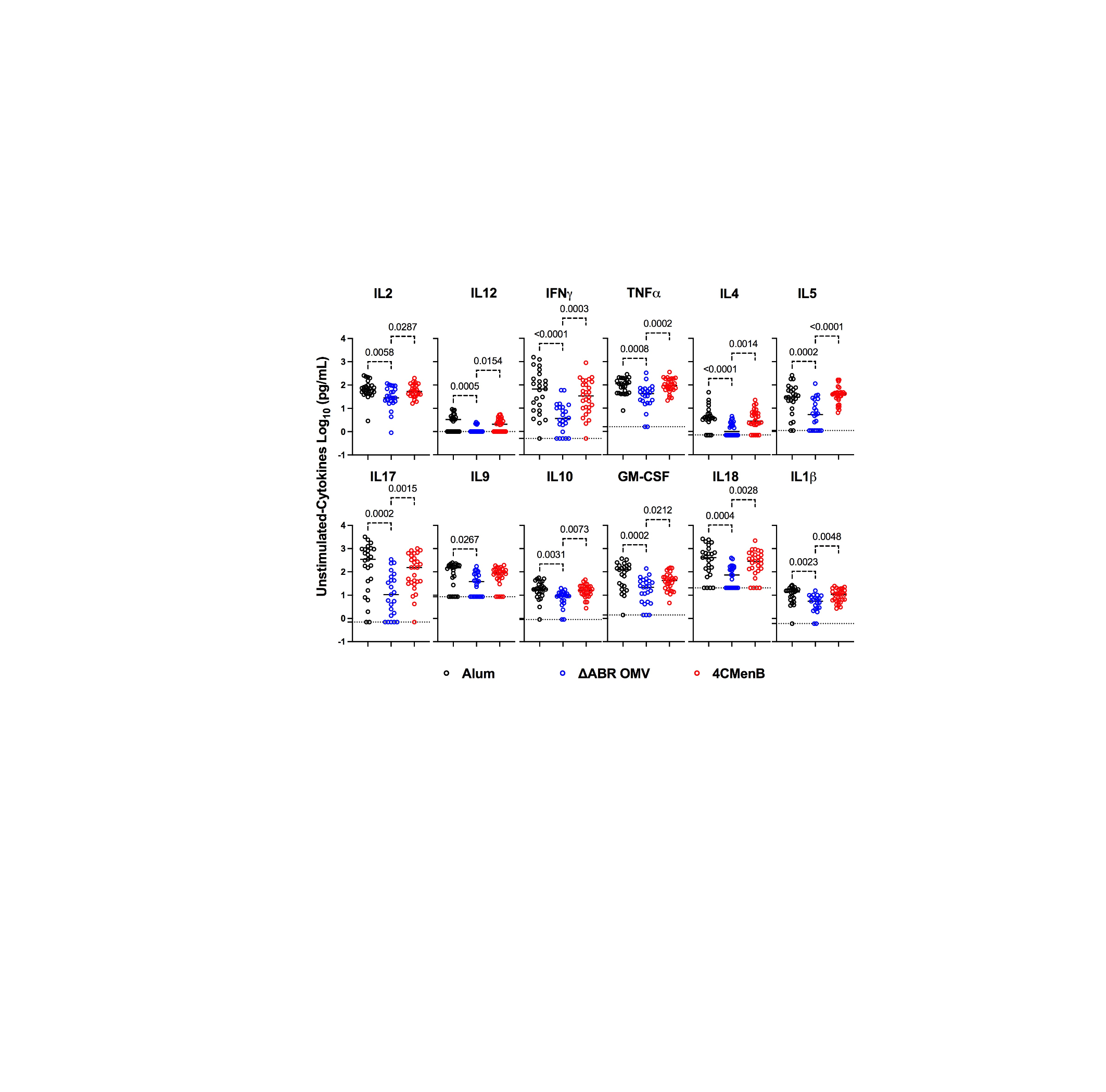


**Supplemental Figure 10**: **Splenocytes from ΔABR immunized mice have diminished secretion of cytokines compared to alum immunized mice.** Comparison of unstimulated (baseline) secreted cytokine/chemokine in culture supernatant from cultured splenocytes from Alum (black), MC58 ΔABR (blue) and 4CMenB (red) immunized mice. Data are shown with symbols showing measured level of the indicated cytokine from splenocytes from and individual mouse; horizontal bar represents the group mean value. Statistical significance was determined using ordinary one-way ANOVA with Tukey’s multiple comparisons, showing p-values only for significant changes.

Created in BioRender. Duncan, J. (2025) <https://BioRender.com/t63v709>

**Supplemental Figure 11**


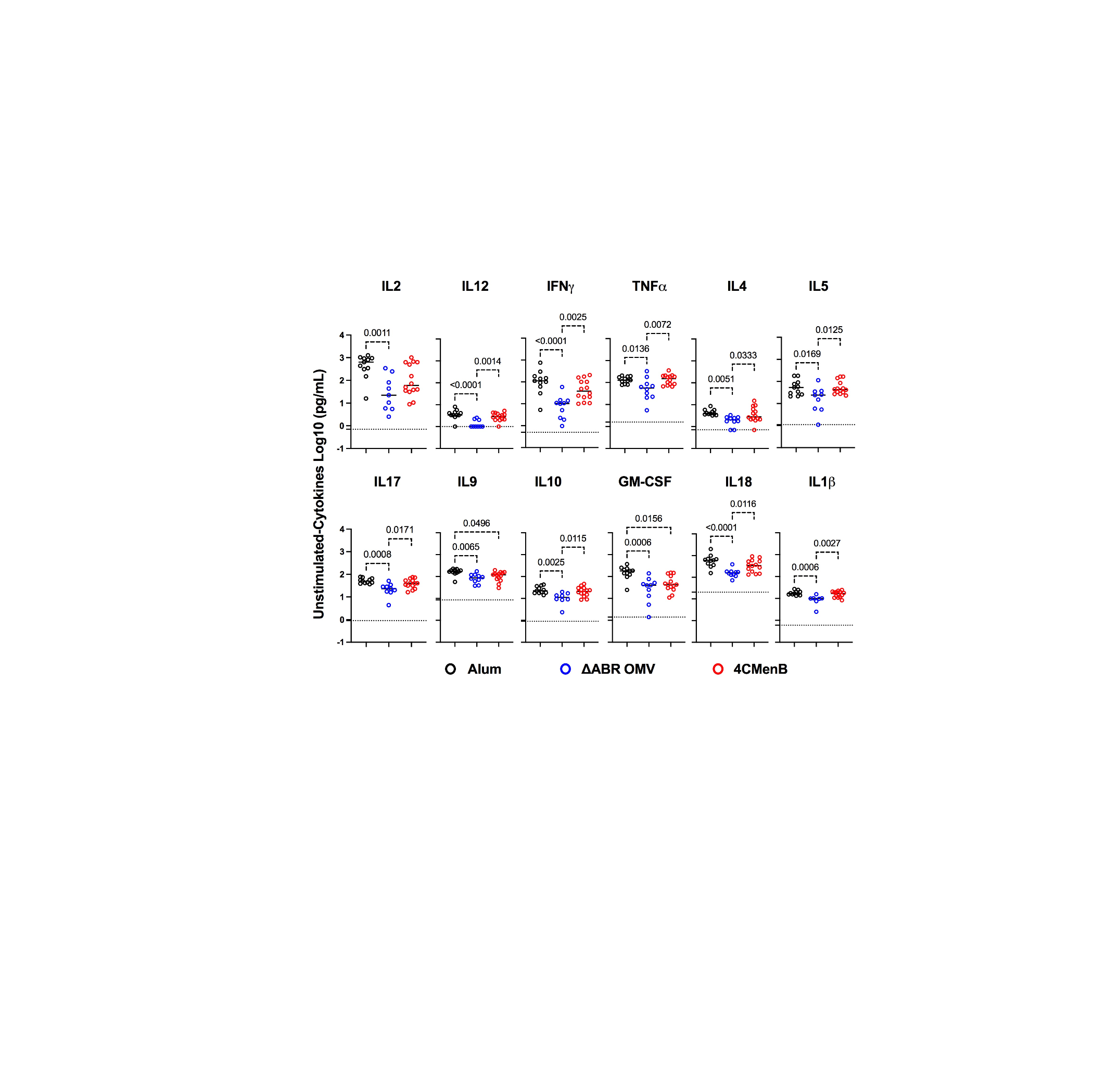


**Supplemental Figure 11**: **Experiment 1: Splenocytes from ΔABR immunized mice have diminished secretion of cytokines compared to alum immunized mice.** The data from the first immunization/challenge experiment performed is presented in this figure. Comparison of unstimulated (baseline) secreted cytokine/chemokine in culture supernatant from cultured splenocytes from Alum (black), MC58 ΔABR (blue) and 4CMenB (red) immunized mice. Data are shown with symbols showing measured level of the indicated cytokine from splenocytes from and individual mouse; horizontal bar represents the group mean value. Statistical significance was determined using ordinary one-way ANOVA with Tukey’s multiple comparisons, showing p-values only for significant changes.

Created in BioRender. Duncan, J. (2025) <https://BioRender.com/z04t528>

**Supplemental Figure 12**

**
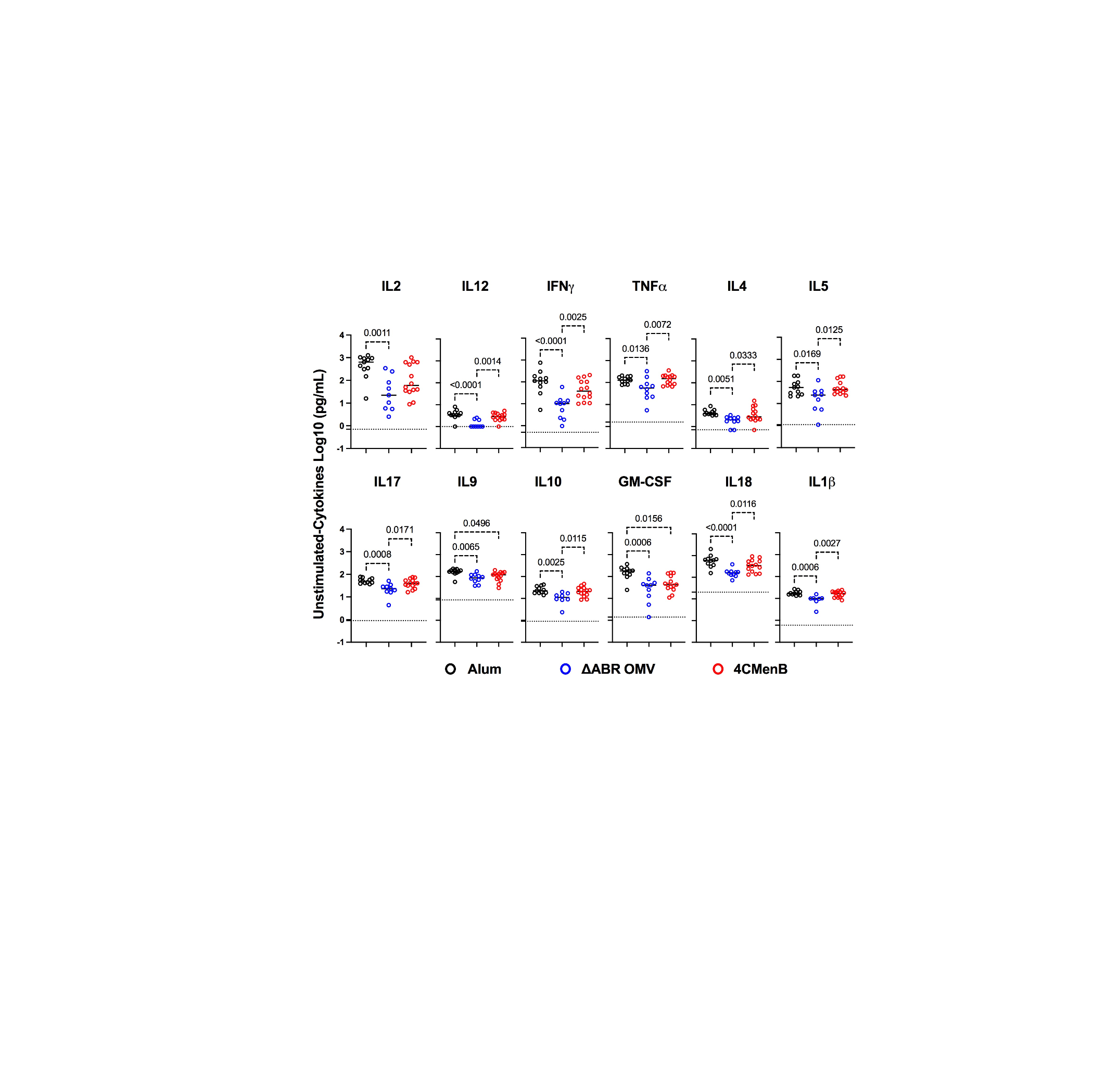
**

**Supplemental Figure 12**: **Experiment 2: Splenocytes from ΔABR immunized mice have diminished secretion of cytokines compared to alum immunized mice.** The data from the second immunization/challenge experiment performed is presented in this figure. Comparison of unstimulated (baseline) secreted cytokine/chemokine in culture supernatant from cultured splenocytes from Alum (black), MC58 ΔABR (blue) and 4CMenB (red) immunized mice. Data are shown with symbols showing measured level of the indicated cytokine from splenocytes from and individual mouse; horizontal bar represents the group mean value. Statistical significance was determined using ordinary one-way ANOVA with Tukey’s multiple comparisons, showing p-values only for significant changes.

Created in BioRender. Duncan, J. (2025) <https://BioRender.com/t74m224>

**Supplemental Figure 13**


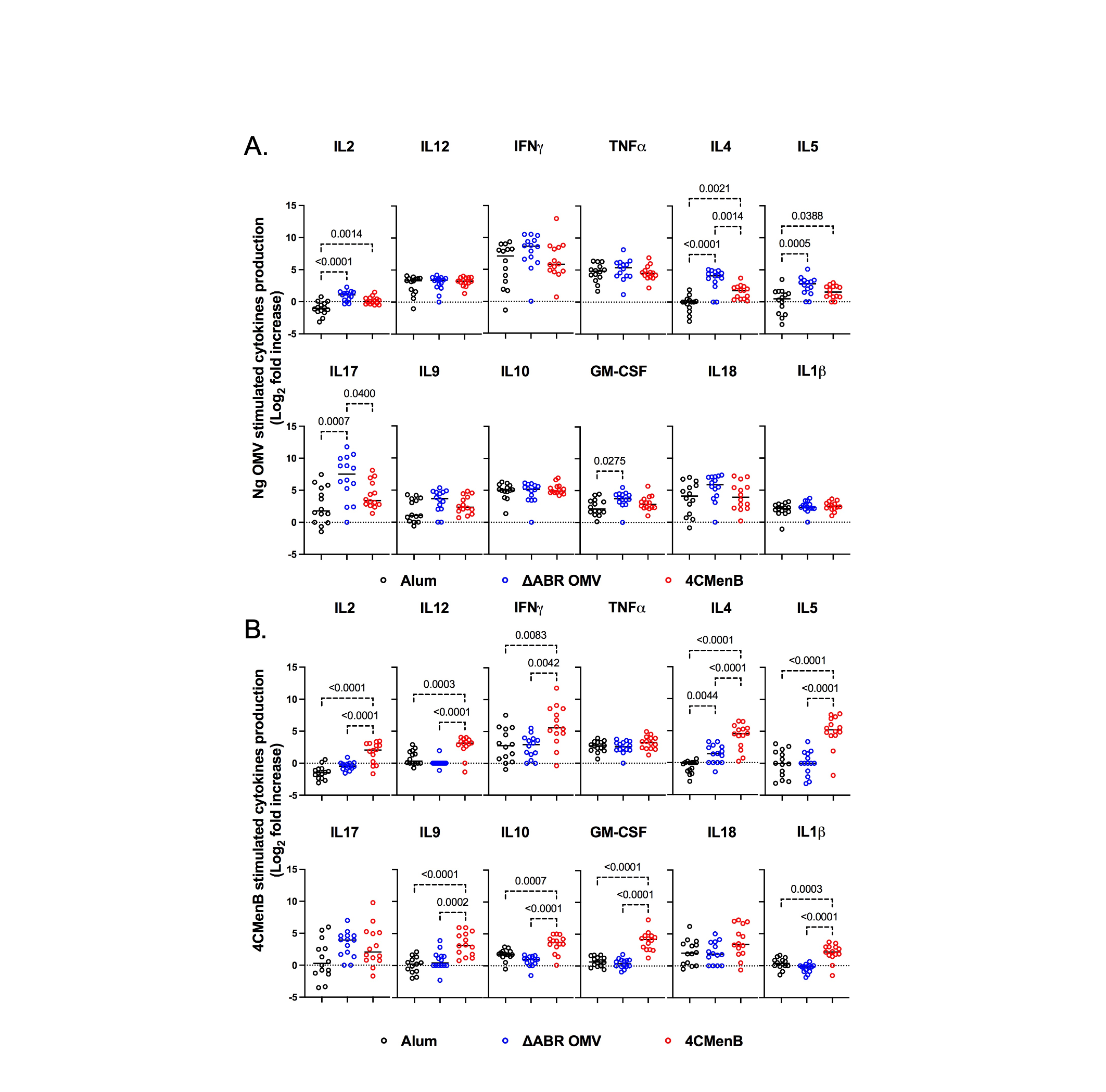


**Supplemental Figure 13: Experiment 1: Immunization with MC58 ΔABR OMV and 4CMenB induce *N. gonorrhoeae* antigen induced cellular immune responses.** The data from the first immunization/challenge experiment performed is presented in this figure. Splenocytes were collected from mice immunized with Alum, MC58 ΔABR, or 4CmenB and subsequent *N. gonorrhoeae* vaginal challenge. Cells were cultured without stimulation or were stimulated ex vivo with either *N. gonorrhoeae* OMV (A) or 4CMenB (B). After 48 hours, cell culture supernatant was collected and the indicated secreted cytokines were measured using a multiplexed bead array based assay. The antigen-induced response is reported as fold increase in cytokine production in supernatant from antigen stimulated cells and unstimulated cells. Statistical significance was performed using one-way ANOVA followed by no paring Šidák’s multiple comparison test, with a single polled variance.

Created in BioRender. Duncan, J. (2025) <https://BioRender.com/y23r867>

**Supplemental Figure 14**

**
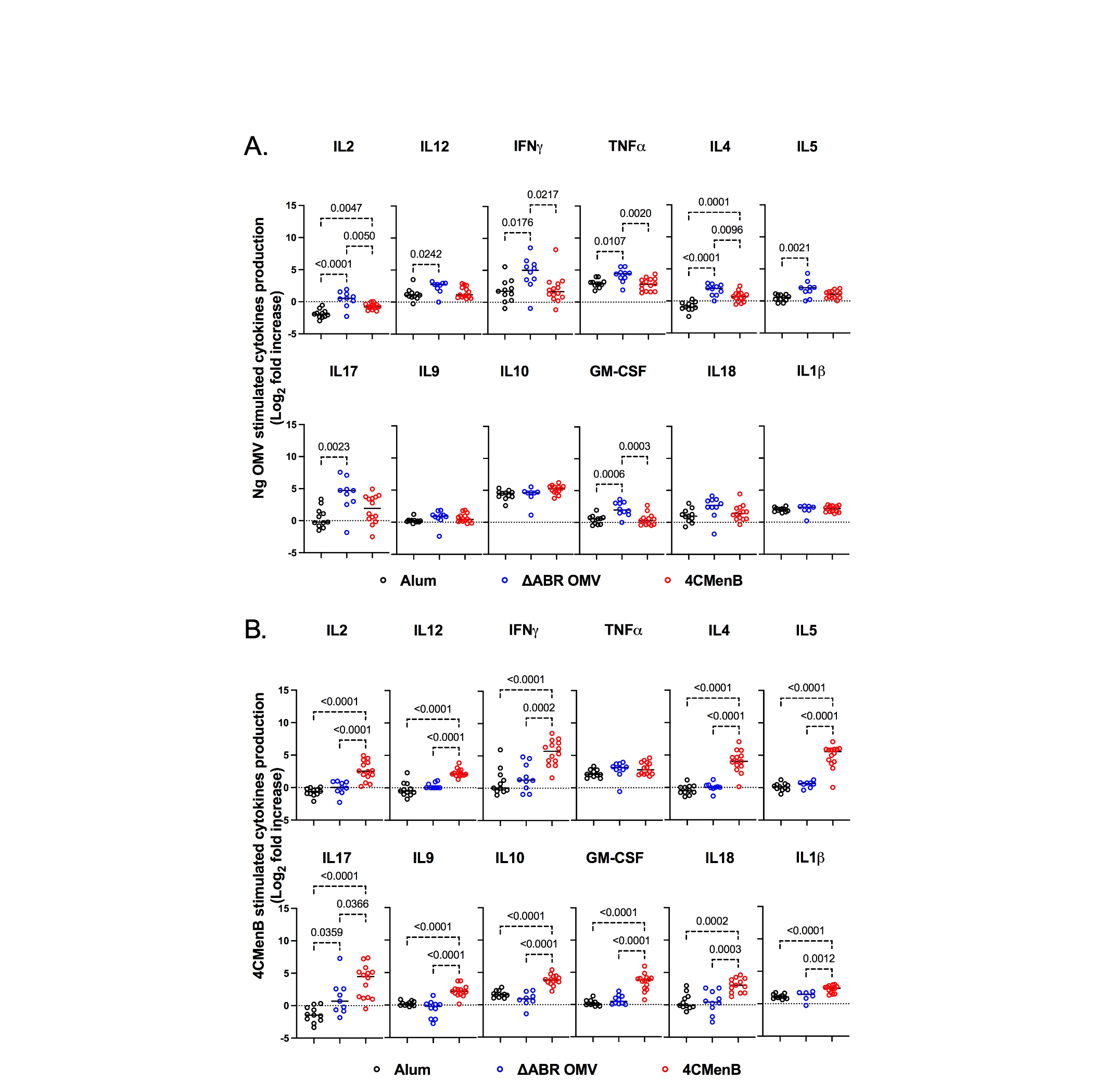
**

**Supplemental Figure 14: Experiment 2: Immunization with MC58 ΔABR OMV and 4CMenB induce *N. gonorrhoeae* antigen induced cellular immune responses.** The data from the second immunization/challenge experiment performed is presented in this figure. Splenocytes were collected from mice immunized with Alum, MC58 ΔABR, or 4CmenB and subsequent *N. gonorrhoeae* vaginal challenge. Cells were cultured without stimulation or were stimulated ex vivo with either *N. gonorrhoeae* OMV (A) or 4CMenB (B). After 48 hours, cell culture supernatant was collected and the indicated secreted cytokines were measured using a multiplexed bead array based assay. The antigen-induced response is reported as fold increase in cytokine production in supernatant from antigen stimulated cells and unstimulated cells. Statistical significance was performed using one-way ANOVA followed by no paring Šidák’s multiple comparison test, with a single polled variance.

Created in BioRender. Duncan, J. (2025) <https://BioRender.com/e11o187>

**Supplement Figure 15**


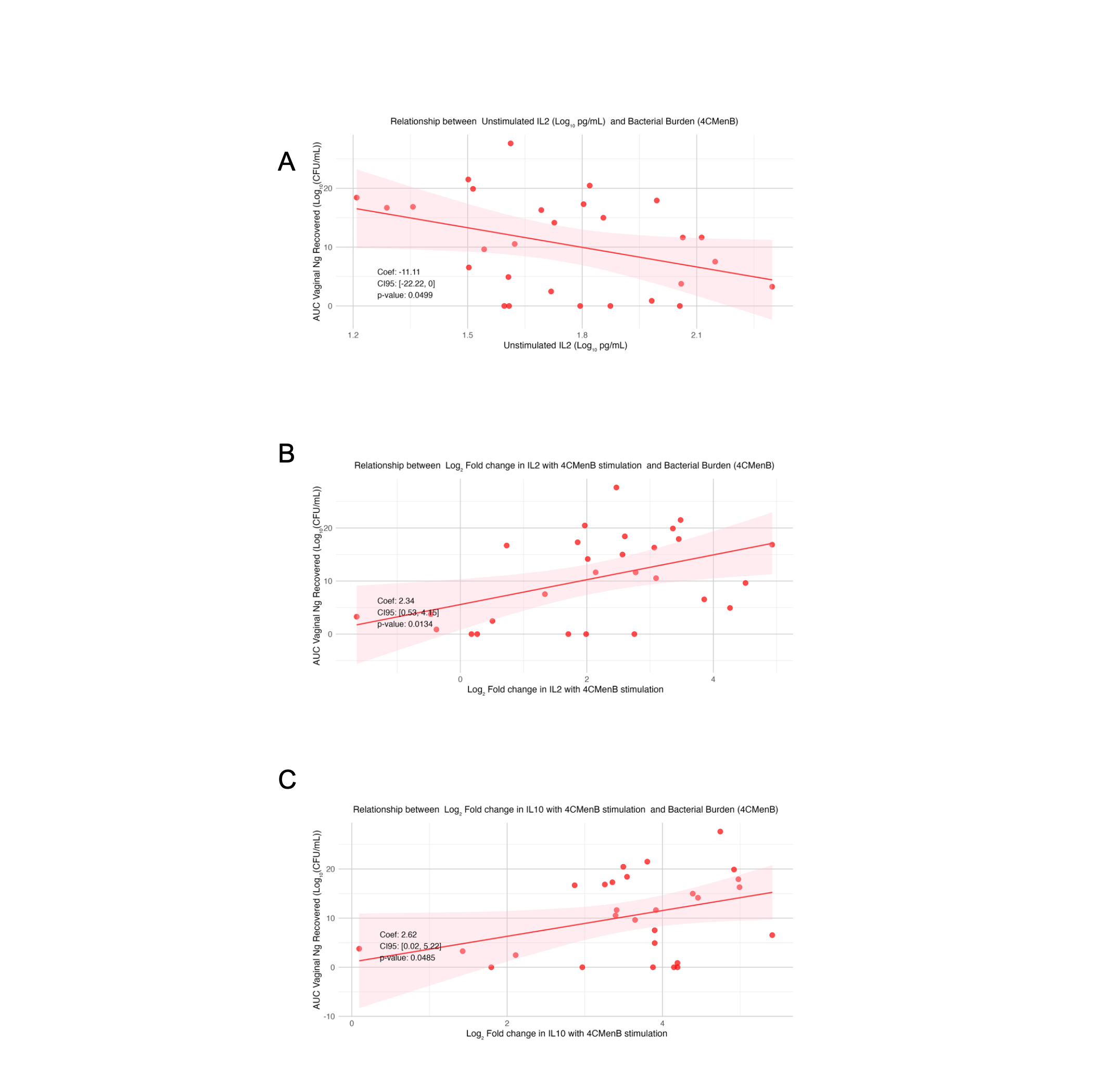


**Supplement Figure 15**: **Linear regression analysis of vaginal *N. gonorrhoeae* burden and antigen stimulated cytokine production.** The relationship between bacterial burden and The relationship between bacterial burden and cytokine markers in the 4CMenB vaccine group was assessed with linear regression in R. Regression coefficients (Coef), 95% confidence intervals (CI95 shown as shaded regions), and p-values are displayed for each panel. The points depict the data points for each mouse. The burden of *N. gonorrhoeae* recovered from vaginal swabs (AUC Log10 CFU/mL) was plotted against the level of IL2 secreted into the supernatant by unstimulated splenocytes collected from immunized mice (Log_2_pg/mL). The burden of *N. gonorrhoeae* recovered from vaginal swabs (AUC Log10 CFU/mL) was plotted against IL2 production by 4CMenB stimulated cells compared to their unstimulated counterparts (expressed as Log_2_ fold change). Similarly, the burden of *N. gonorrhoeae* recovered from vaginal swabs (AUC Log10 CFU/mL) was plotted against the Log2 fold change in IL10 production by stimulated cells compared to their unstimulated counterparts was also correlated with higher bacterial burden. Created in BioRender. Duncan, J. (2025) <https://BioRender.com/p89e661>
